# A Last Resort When There is No Blood: Experiences and Perceptions of Intraoperative Autotransfusion Among Medical Doctors Deployed to Resource-Limited Settings

**DOI:** 10.1007/s00268-020-05749-y

**Published:** 2020-08-27

**Authors:** Annie Sjöholm, Andreas Älgå, Johan von Schreeb

**Affiliations:** Department of Global Public Health, Karolinska Institutet, 171 77 Stockholm, Sweden

## Abstract

**Background:**

Four and a half million people die globally every year due to traumatic injuries. One major cause of preventable death is bleeding. Blood for transfusion is often unavailable in resource-limited settings, where a majority of trauma deaths occur. Intraoperative autotransfusion (IAT) has been proposed as a safe and feasible lifesaving alternative to allogeneic blood transfusion. However, there is limited knowledge regarding its use among doctors working for international non-governmental organisations (INGOs) in resource-limited settings. The aim of this study was to explore the experiences and perceptions of IAT among INGO-affiliated medical doctors with clinical experience in resource-limited settings.

**Methods:**

We conducted semi-structured interviews via telephone or Skype with 12 purposefully sampled surgeons and anaesthesiologists. The interviews were recorded, transcribed verbatim, and analysed using content analysis.

**Results:**

We identified three main themes relating to IAT and bottlenecks preventing the scale-up of its use: variation in techniques and systems, contextual factors, and individual medical doctor factors. The participants gave detailed reports of missed opportunities for usage of IAT in resource-limited settings. Bottlenecks included the lack of simple and cost-effective products, limited availability of protocols in the field, and insufficient knowledge and experience of IAT.

**Conclusions:**

The participants found that simple IAT is under-utilised in resource-limited settings. Missed opportunities to use IAT were mainly associated with armed conflict settings and obstetrical emergencies. In order to meet the need for IAT in resource-limited settings, we suggest further consideration of the identified bottlenecks.

## Introduction

Globally, an estimated four and a half million people die every year due to traumatic injuries [1]. Most deaths and disabilities resulting from traumatic injury occur in low- and middle-income countries where healthcare capacities are constrained by limited resources. In many resource-limited settings, conflicts and disasters add to the trauma care burden [2]. One major cause of preventable death is bleeding [3, 4], which could be prevented by blood transfusion [5]. However, in resource-limited settings—especially following disasters—blood for transfusion remains scarce [6]. In addition, the safety of allogeneic blood transfusion is not guaranteed in these settings, often because of a high prevalence of blood-borne infections and a limited ability to test blood products [7]. Alternatives to allogeneic blood transfusion are therefore a high priority [8].

Intraoperative autotransfusion (IAT) is an established and safe technique in which blood from a bleeding patient is collected and reinfused into the same patient [9]. IAT has been shown to reduce the need for allogeneic blood [9–12]. In high-income countries (HICs), advanced device-based IAT is used to collect and reinfuse washed red blood cells, primarily in planned surgeries [13, 14]. The device-based IAT requires supplies that are not routinely available in resource-limited settings.

Simplified variations of IAT are a well-established alternative for use in resource-limited settings, described for patients with haemothorax, ruptured ectopic pregnancy, and injuries to the liver or spleen [15–17]. These simple IAT techniques begin with blood collection, which is performed by either an open system using a gallipot or soup ladle, or by a closed system using a special collecting device or a chest tube, followed by reinfusion, with or without filtration [18]. Simple IAT has been shown to be well accepted by local medical doctors in life-threatening situations [13]. A major advantage of simple IAT, compared to allogeneic blood transfusion, is the reduced time until transfusion because there is no need for cross-matching the blood [13]. Disadvantages include the costs of educating the staff and using consumables for the equipment (Table 1) [19].

**Table 1:** Advantages and disadvantages of the use of simple intraoperative autotransfusion compared to allogeneic blood transfusion

Advantages	Disadvantages
Shortens the time to transfusion	Requires experienced operators
Reduces the need for allogeneic blood transfusion	Risks inducing coagulopathy and disseminated intravascular coagulation
Lowers the risk of transfusion-transmitted infections and transfusion reaction	Associated expenditures for equipment and staff training
Increases the blood availability for patients with rare blood groups and multiple autoantibodies	

International non-governmental organisations (INGOs) regularly provide emergency surgical care in resource-limited settings, especially following disasters and during armed conflict. INGOs typically have more resources than the national health system and are often staffed with experienced international staff from HICs. Deployed medical personnel from INGOs are trained in HICs and may therefore be unfamiliar with the equipment and techniques adapted for resource-limited settings [20]. There is a paucity of knowledge regarding the utility of IAT in resource-limited settings and the bottlenecks preventing the scale-up of its use [14]. We therefore aimed to explore the experiences with and perceptions of simple IAT among deployed surgeons and anaesthesiologists from HICs working in resource-limited settings.

## Materials and methods

### Participants

We purposefully selected surgeons and anaesthesiologists with substantial experience working for INGOs in resource-limited settings. We chose to interview surgeons and anaesthesiologists because they are in command during operations and they decide whether it is necessary to perform IAT. To ensure that only key experts were selected, participants had to be specialists in either anaesthesiology or surgery, trained in HICs, with more than three years of clinical experience in resource-limited settings. Background information on the participants is presented in Table 2.

**Table 2:** Characteristics of the 12 participants

Characteristic	*n*/years
Sex, *n*	
Female	6
Male	6
Age, years	
Median	52
Interquartile range	42–54
Medical specialty, *n*	
Surgeon	6
Anaesthesiologist	6
Working experience in resource-limited settings, years	
Median	10
Interquartile range	8–18
Experience with simple intraoperative autotransfusion, *n*	
In resource-limited settings	10
In high-resource settings only	2

### Data collection

The research team developed an interview guide (“Appendix 1”), and an experienced qualitative researcher reviewed the guide to optimise relevance and design. One author (A.S.) conducted semi-structured individual interviews in either Swedish or English via telephone or Skype in October 2018. Some adjustments were made to the guide in between interviews. Twelve medical doctors were invited to participate, and all consented to the study. The duration of the interviews ranged from 10 to 49 min with a mean duration of 23 min.

Each participant was given an identification code. The identification codes were safeguarded at the research facility for the duration of the study.

### Data analysis

One author (A.S.) transcribed the interviews verbatim and read the interview material in its entirety repeatedly to fully grasp the content. Subsequently, we coded and analysed the data as a group in accordance with content analysis, as described by Graneheim and Lundman [21]. The analysis was inductive with manifest and latent content analysis. We identified meaning units, which we condensed and labelled with codes. We then categorised and summarised the codes into subcategories. Subsequently, we abstracted the subcategories and sorted them into categories, from which we derived themes. An inductive thematic saturation approach was used, meaning that we found the material to be saturated when additional data did not lead to the emergence of new codes or themes [22]. We used the table tool in Microsoft Word® 2018 (Microsoft, Redmond, Washington, USA) for the analysis. Quotations from interviews conducted in Swedish were translated into English. We conducted member checks of the analytical results with all participants. An example of the abstraction from meaning unit to category is shown in Table 3.

**Table 3:** Example of abstraction from meaning unit to category

Main category	Subcategory	Code	Condensed meaning unit	Meaning unit
Usability is limited to certain settings	Armed conflict context with thorax and abdominal trauma	Usage of intraoperative autotransfusion is suitable for traumatic injuries	Intraoperative autotransfusion was used at a few occasions in patients with fulminant haemothorax	When they had such fulminant haemothorax that the circulation failed, we would give transfusion, I know that on a few occasions the blood that came out of the thoracic drainage was used
The physician’s experience of intraoperative autotransfusion in the field	Missed opportunities	The wasted shed blood could be a valuable resource	In obstetrical emergencies, the shed blood is thrown out. The blood could have been used for autotransfusion	They opened the woman and threw all the blood; it was around 1.5 L…. But the woman definitely should have been saved if we could have given her, her own blood

### Trustworthiness

To improve trustworthiness, we conducted thorough discussions of the categorisation with an experienced qualitative researcher. We discussed differences in codes and categories until consensus was reached. We present representative quotations extracted from the original material to demonstrate our reasoning and to facilitate the reader in judging the credibility and authenticity of our findings.

### Ethical considerations

This study was conducted in accordance with the Declaration of Helsinki. Participation was voluntary, and verbal informed consent was obtained before conducting the interviews. Participants were informed that they could withdraw from the interview at any time and they were not offered any incentives. Ethical permission was not sought as the research team determined that this study did not include sensitive personal information. All participants formally approved publication after the results of the study were presented to them.

## Results

Three themes emerged in the content analysis: variation in techniques and systems, contextual factors, and individual medical doctor factors. The material was divided into a total of six categories and 17 subcategories. Each theme emerged from two categories. A summary of the themes, categories, and subcategories is presented in Table 4.

**Table 4:** Themes, categories, and subcategories relating to simple intraoperative autotransfusion based on interviews with key experts from resource-limited settings

Theme	Category	Subcategory
Variation of technique	Different techniques due to differences in situation and protocols	Different techniques for different indications
		Indications differ between protocols
		Controversy on the usage of bowel contaminated blood
	Different systems in different settings	Commercial system built for high-resource settings
		Predefined self-built system
		Improvised self-built system
Contextual factors	Usability is limited to certain areas	Armed conflict context with thorax and abdominal trauma
		Obstetric emergencies
		Different opinions on the need in humanitarian resource-limited settings
	Bottlenecks related to the setting	Lack of a simple and cost-effective product
		Limited availability of protocols in the field
		Patients arriving late to hospital, in severe shock or dead on arrival
Individual medical doctors’ factors	The medical doctors’ experience of intraoperative autotransfusion in the field	Low usage rate
		Missed opportunities
	Bottlenecks related to the medical doctors	Insufficient knowledge
		Limited experience
		Limited experience of preparation of equipment

A description of the themes is presented below, along with anonymised quotations. The number following the quotation is the participant identification code.

### Variation in techniques and systems

This theme focuses on the techniques and systems and was built from two categories: different techniques resulting from differences in situation and protocols, and different systems in different settings. The latter category focuses on how the techniques are influenced by the context, as opposed to the context itself. The IAT technique had been taught to the surgeons and anaesthesiologists by colleagues in the field. The details of the IAT technique differed with regard to the number of layers of gauze used for filtration and the addition of anticoagulants. Participants highlighted that it is not necessary to add anticoagulants to blood collected from a haemothorax, based on their own experience. An explanation for the variation of technique was that it is largely experienced based.

The participants described how different systems for IAT are used depending on whether they are located in HICs, low-income countries, or armed conflict settings. Improvised self-built systems for IAT were described to have been used in armed conflict settings when IAT was not prepared in the operating room and patients were in need of blood. In situations with insufficient preparation for IAT in the operating room or a lack of equipment, participants described using coffee filters or the blood bag itself for filtration before reinfusing the blood.

Participants stated that there is controversy regarding the use of IAT in cases where blood might be contaminated by bowel content. A minority of the interviewed surgeons and anaesthesiologists had experience with IAT in patients with intestinal contamination. The use of IAT for patients in this category was described as a last resort for those who otherwise would have died of anaemia.

I knew that the patient would die on the operating table because there was no other source of blood, there was no donor, and it was a last resort. (9).

Interestingly, the participants who had used IAT in these cases were hesitant to use it again because they had been criticised by colleagues for their actions. As infusion of contaminated blood could induce sepsis, IAT with potentially contaminated blood was seen as a high-risk procedure. Therefore, the participants questioned the use of IAT when blood might be contaminated by bowel content.

### Contextual factors

This theme focuses on contextual factors and was constructed from two categories: usability being limited to certain areas, and bottlenecks related to the setting. The participants believed there was a need for IAT in areas of armed conflict and obstetrical emergencies. IAT was considered beneficial in armed conflict for massive haemothorax because it is a quick treatment and easy to learn. Participants had experienced missed opportunities when IAT could have been used; some described situations during obstetrical emergencies when blood from the patient had been wasted, even though there was no allogeneic blood available. The common denominator for the use of IAT was the massive blood loss that results in life-threatening conditions.

You come with a patient with a lot of blood and we unfortunately, we throw it away. (2).

However, the participants had some concerns regarding the implementation of the technique in certain contexts, mainly related to prioritisation.

Hemorrhagic shock with ectopic pregnancies not, like, every day, so I think that there are not so many indications. So, the energy we will put into this process … for me would be better used to set up complete blood bank where we can. (3).

If you think about having to get the equipment, create the protocol, train the surgeons to do it. Yeah, among the other things that we could be training them to do. So, we would prioritise probably other things, like, for example, ultrasound scanning. (12).

One of the potential bottlenecks described was the lack of a simple and cost-effective product. The participants explained that the open system IAT technique used for abdominal haemorrhage was laborious for the personnel involved. This technique involves collecting the blood with a soup ladle and filtering it through several layers of gauze before reinfusion. Some of the surgeons expressed a need for a simple product for IAT since the open IAT technique takes focus away from the surgery. Both surgeons and anaesthesiologists admitted that the complications of the open IAT technique resulted in less usage, leading to a knowledge gap about when to use open IAT. In addition, it was believed that there were missed opportunities secondary to the scarcity of protocols in the hospitals. A majority of the interviewed doctors highlighted that there are no protocols for IAT in abdominal injuries and obstetrical cases, while protocols for haemothorax do exist.

I think it’s an underused tool, it could be used more often, because in my experience when I've been out… it's used quite rarely. (4).

### Individual medical doctor factors

This theme was built from two categories: the medical doctors’ experiences of IAT in the field, and bottlenecks related to the medical doctors. The participants expressed that IAT has a low usage rate within INGOs. This was believed to be a result of limited knowledge and experience with IAT. Inadequate research on IAT in resource-limited settings was one explanation for the lack of knowledge. Participants believed that there is a significant imbalance between IAT experiences and scientific publications.

Another explanation given was that insufficient knowledge among policy makers and senior clinicians resulted in protocols not being updated. Participants explained that a majority of policy makers had received their medical training in HIC and therefore had been given limited education on IAT techniques suitable for resource-limited settings. Consequently, the lack of awareness among policy makers risks negatively affecting the availability and use of IAT. The participants stated that deployed medical doctors were aware of the use of IAT in places where the senior clinicians had knowledge of it and promoted it.

Lack of knowledge among those who work today in the humanitarian organisation, the policy makers, the senior clinicians. (5).

## Discussion

The interviewed surgeons and anaesthesiologists with previous deployment by INGOs to resource-limited settings described a low usage rate of IAT within INGOs, mainly related to limited knowledge and experience with the technique. Nonetheless, the participants expressed that IAT could be lifesaving in situations when no allogeneic blood is available. The main bottleneck for wider use was limited knowledge and experience performing simple IAT techniques. The participants, for example, described missed opportunities to use IAT for obstetrical emergencies. In sub-Saharan Africa, one out of four maternal haemorrhage deaths has been found to be due to a lack of blood transfusion possibilities [23]. An explanation offered by the participants for the missed opportunities presented in the current study was the limited availability of IAT protocols in INGO surgery clinics. This explanation is in line with previously published findings that IAT in trauma patients is underused due to limited blood transfusion policies [11]. One of the reasons for the poor knowledge of the technique according to the participants was the deployed medical doctors’ background in HICs. Deployed medical doctors were perceived as having limited knowledge about the self-built method because of the differences in healthcare systems in resource-limited settings relative to HICs.

One important bottleneck to wider usage of simple IAT was found to be the lack of a simple and cost-effective product. The participants stated that the self-built IAT system for abdominal bleeding was laborious, and because of its complexity, underused, which is in line with previous research [17].

Participants described experiences of IAT using blood potentially contaminated by bowel content that resulted in positive outcomes. There is controversy surrounding this practice, but studies have found that IAT might be safely used when blood could be contaminated by bowel content [12, 24–26]. It should be noted that the scope of the present study differs from previous studies because we discuss self-built systems as opposed to commercial devices. Our findings suggest that more research on the safety of IAT with potentially contaminated blood is needed.

This study has a number of limitations. First, the method involves the risk of generating bias because the researcher is the primary instrument of data collection. To decrease researcher bias, all authors were actively involved in the content analysis, aiming for consensus in interpretation and coding. In addition, we consulted an experienced qualitative researcher regarding the categorisation. Second, English is not the native language of either the authors or the majority of the participants. However, English is the language used by the participants in the workplace, so the risk of language influencing the results was considered negligible. Finally, the qualitative methodology and the low number of participants limit generalisability. However, we aimed to achieve a heterogeneous group, selecting male and female surgeons and anaesthesiologists with substantial clinical experience from two different INGOs in resource-limited settings which may have increased the transferability to expatriate medical doctors in similar settings [27].

Simple IAT is a promising technique. However, practical protocols and further studies are needed to ensure adequate use. Protocols for IAT use in patients with abdominal injuries and acute obstetrical conditions should be developed and promoted. Our results provide new aspects for consideration when medical doctors are preparing for deployment and may help to further improve the treatment of injuries to the thorax or abdomen in resource-limited settings. Future research could focus on the experiences and perceptions of IAT among local medical doctors in resource-limited settings.

## Conclusions

The participants described that simple IAT is under-utilised in resource-limited settings. Missed opportunities to use IAT were mainly found in armed conflicts and obstetrical emergencies. Bottlenecks included the lack of simple and cost-effective products, limited availability of protocols in the field, and insufficient knowledge and experience of IAT. To meet the need for IAT in resource-limited settings, these bottlenecks must be considered.

## Appendix 1: Intraoperative autotransfusion interview guide

1. Do you have personal experience from using intraoperative autotransfusion (IAT) in a low-resource setting, please elaborate?
  Probe: blood collection, filtration, clotting, air embolism, contamination, anticoagulant, reinfusion, allogeneic blood supply, similar or different compared to a typical case
2. Have you ever been in a situation where IAT could have been used during your deployments, please elaborate?
  Probe: situation, blood supply, personnel, knowledge, guidelines, equipment
3. For what patients would you consider using IAT in a low-resource setting?
  Probe: obstetrics (ectopic pregnancies), trauma (haemothorax, blunt abdominal), orthopaedic (femur fracture)
4. What is your view on the use of IAT by organisations like yours in a low-resource setting?
  Probe: protocols, acceptable technique, promoted technique, simplified techniques, new devices, area of use, future use
5. What do you consider are the main bottlenecks that limit a wider use of IAT?
  Probe: personnel, knowledge, equipment, cost, guidelines
6. Is there something you would like to add?

### General information

1. Sex?
2. Age?
3. Title/specialty?
4. Which organisation are you currently working for?
5. Position?
6. Year of medical degree?
7. Year of specialisation?
8. Years of experience in humanitarian/low-resource settings?

## References

[1] Roth GA, Abate D, Abate KH (2018). Global, regional, and national age-sex-specific mortality for 282 causes of death in 195 countries and territories, 1980–2017: a systematic analysis for the global burden of disease study 2017. The Lancet.

[2] World Health Organization, International Society of Surgery, International Association for the Surgery of Trauma and Surgical Intensive Care (2004). Guidelines for essential trauma care.

[3] World Health Organization (2010) Injuries and violence: the facts

[4] Cothren C, Moore E, Hedegaard H (2007). Epidemiology of urban trauma deaths: a comprehensive reassessment 10 years later. Off J Int Soc Surg.

[5] Coupland R (1996). Abdominal wounds in war. Br J Surg.

[6] World Health Organization (2017). Global status report on blood safety and availability 2016.

[7] Roberts DJ, Field S, Delaney M (2016). Problems and approaches for blood transfusion in the developing countries. Hematol Oncol Clin North Am.

[8] Osaro E, Charles AT (2011). The challenges of meeting the blood transfusion requirements in Sub-Saharan Africa: the need for the development of alternatives to allogenic blood. J Blood Med.

[9] World Health Organization (2001). Blood transfusion safety team the clinical use of blood: handbook.

[10] Carless PA, Henry DA, Moxey AJ (2010). Cell salvage for minimising perioperative allogeneic blood transfusion. Cochrane Database Syst Rev.

[11] Rhee P, Inaba K, Pandit V (2015). Early autologous fresh whole blood transfusion leads to less allogeneic transfusions and is safe. J Trauma Acute Care Surg.

[12] Bowley DM, Barker P, Boffard KD (2006). Intraoperative blood salvage in penetrating abdominal trauma: a randomised, controlled trial. World J Surg.

[13] Selo-Ojeme DO, Onwude JL, Onwudiegwu U (2003). Autotransfusion for ruptured ectopic pregnancy. Int J Gynaecol Obstet.

[14] Brown CR, Foulkrod KH, Sadler HT (2010). Autologous blood transfusion during emergency trauma operations. Arch Surg.

[15] Baldan M, Giannou CP, Rizzardi G (2006). Autotransfusion from haemothorax after penetrating chest trauma: a simple, life-saving procedure. Trop Doct.

[16] Gourlay T, Simpson C, Robertson CA (2018). Development of a portable blood salvage and autotransfusion technology to enhance survivability of personnel requiring major medical interventions in austere or military environments. J R Army Med Corps.

[17] Priuli G, Darate R, Perrin RX (2009). Multicentre experience with a simple blood salvage technique in patients with ruptured ectopic pregnancy in Sub-Sahelian West Africa. Vox Sang.

[18] Giannou C, Baldan M (2013). Molde a war surgery: working with limited resources in armed conflict and other situations of violence.

[19] Walunj A, Babb A, Sharpe R (2006). Autologous blood transfusion. Contin Educ Anaesth Crit Care Pain.

[20] Asgary R, Junck E (2013). New trends of short-term humanitarian medical volunteerism: professional and ethical considerations. J Med Ethics.

[21] Graneheim UH, Lundman B (2004). Qualitative content analysis in nursing research: concepts, procedures and measures to achieve trustworthiness. Nurse Educ Today.

[22] Saunders B, Sim J, Kingstone T (2018). Saturation in qualitative research: exploring its conceptualization and operationalization. Qual Quant.

[23] Bates I, Chapotera GK, McKew S (2008). Maternal mortality in sub-Saharan Africa: the contribution of ineffective blood transfusion services. BJOG : An Int J Obstet Gynaecol.

[24] Timberlake GA, McSwain NE (1988). Autotransfusion of blood contaminated by enteric contents: a potentially life-saving measure in the massively hemorrhaging trauma patient?. J Trauma.

[25] Glover JL, Smith R, Yaw PB (1978). Autotransfusion of blood contaminated by intestinal contents. JACEP.

[26] Esper SA, Waters JH (2011). Intra-operative cell salvage: a fresh look at the indications and contraindications. Blood Transfus.

[27] Graneheim UH, Lindgren B-M, Lundman B (2017). Methodological challenges in qualitative content analysis: a discussion paper. Nurse Educ Today.

